# Supplementary material for: Yersinia entomophaga Tc toxin is released by T10SS-dependent lysis of specialized cell subpopulations
Source: Nat Microbiol. 2024 Jan 18;9(2):390–404. doi: 10.1038/s41564-023-01571-z (PMC10847048; doi:10.1038/s41564-023-01571-z)
Supplement: Supplementary file 1 — Supplementary Figs. 1–5, Table 1 and Supplementary Data references. [file 41564_2023_1571_MOESM1_ESM.pdf]

nature microbiology

Article

https://doi.org/10.1038/s41564-023-01571-z

Yersinia entomophaga Tc toxin is released by T10SS-dependent lysis of specialized cell subpopulations

In the format provided by the authors and unedited

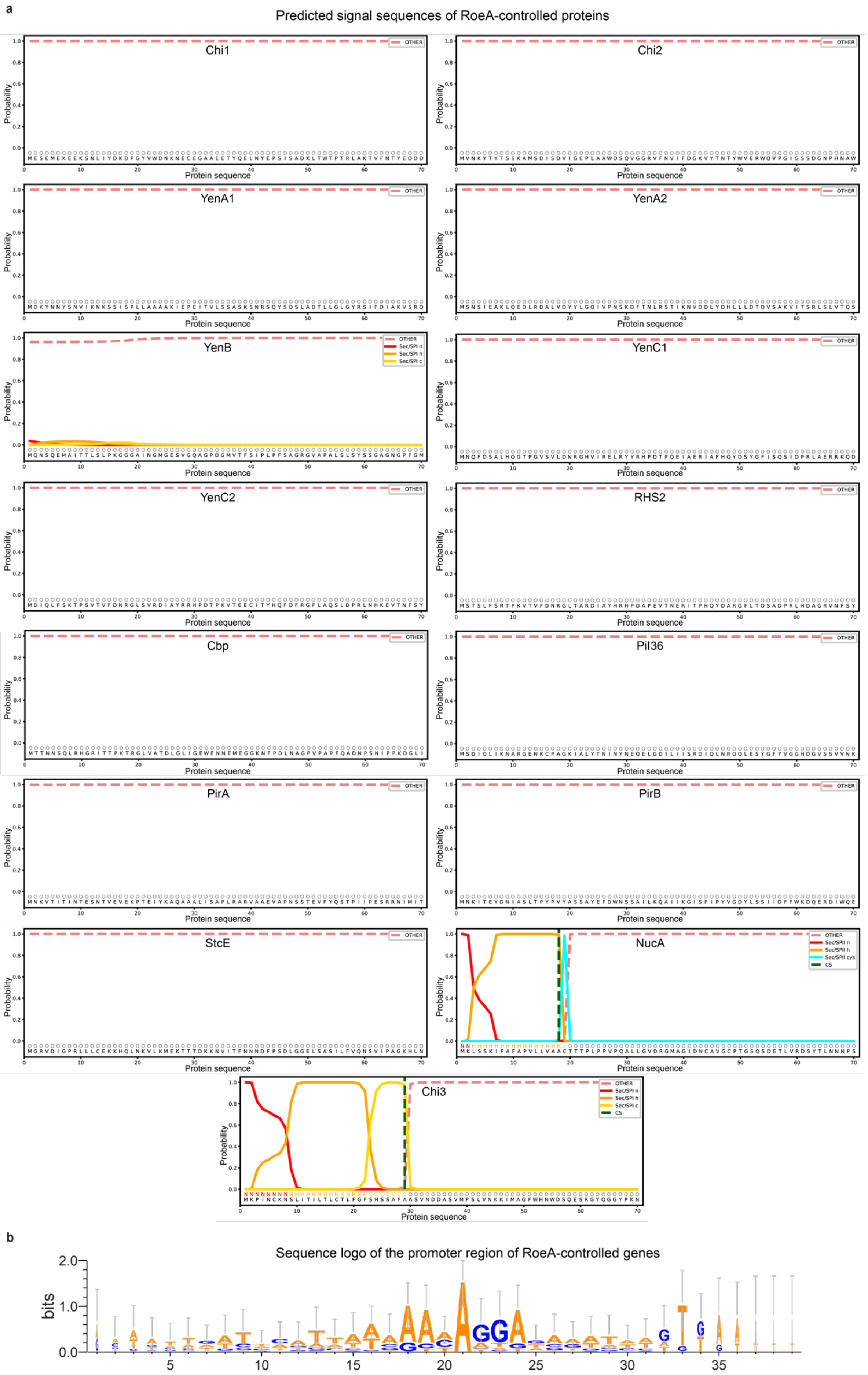
a
Predicted signal sequences of RoeA-controlled proteins
Chi1
Chi2
YenA1
YenA2
YenB
YenC1
YenC2
RHS2
Cbp
Pil36
PirA
PirB
StcE
NucA
Chi3
Probability
Protein sequence
OTHER
Sec/SPI n
Sec/SPI h
Sec/SPI c
Sec/SPII n
Sec/SPII h
Sec/SPII cys
CS
b
Sequence logo of the promoter region of RoeA-controlled genes
bits

Supplementary Figure 1 | Nearly all RoeA-controlled toxins and virulence factors lack an established signal sequence and have a putative RoeA promoter recognition sequence. a, Predictions of secretion signal sequences for RoeA-controlled toxins and virulence factors show that nearly all are unable to utilize alternative export pathways due to lack of a secretion signal sequence. Of those that do, the nuclease NucA contains disulfide bridges that require an oxidative environment for maturation, and periplasmically localized chitinases (as likely the case for Chi3) have been suggested to target soluble oligosaccharides that enter the cell through porins[1]. The relevant UniProt accession numbers are provided in the Methods section. **b,** Sequence logo derived from the promoter region of RoeA-controlled toxins, virulence factors and the YenLC structural component operon indicates an overrepresented sequence that may function as a RoeA binding site.

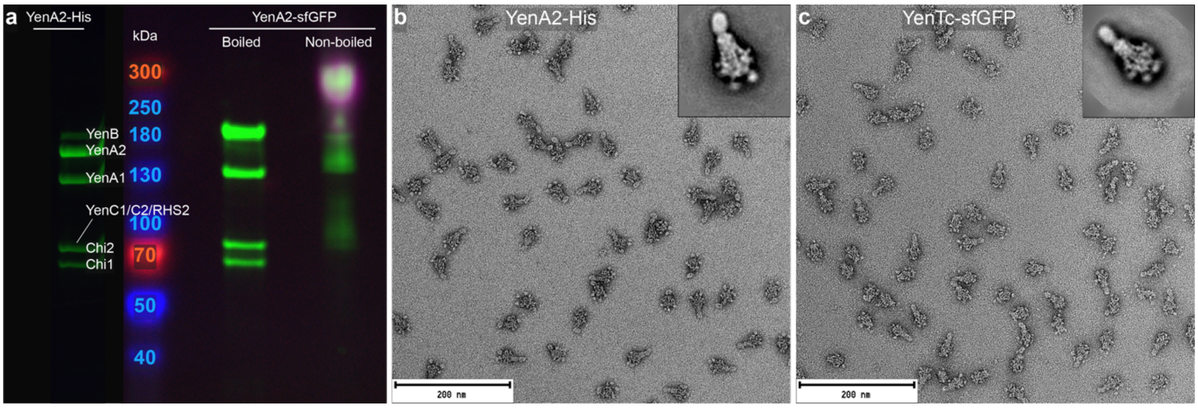

Supplementary Figure 2 | Characterization of purified YenA2-His and YenA2-sfGFP. a, Purified YenA2-His and YenA2-sfGFP samples, with the C-terminal fusion of sfGFP to YenA2 causing the corresponding 156 kDa band to shift upwards. Autoproteolytic cleavage of the 32 kDa C-terminal HVRs causes the band of the 107 kDa YenC1/C2/RHS2 components to conversely shift downwards. The fluorescence signal from the non-boiled YenA2-sfGFP sample is shown as a purple overlay. A mass spectrometric analysis of the YenA2-His sample for experimental validation of its subunit composition is available in the Source Data file. Data is consolidated. n = 3 biological replicates. **b-c,** Negative stain micrographs and class averages (insets) of the YenA2-His and YenA2-sfGFP samples, respectively. n = 3 biological replicates.

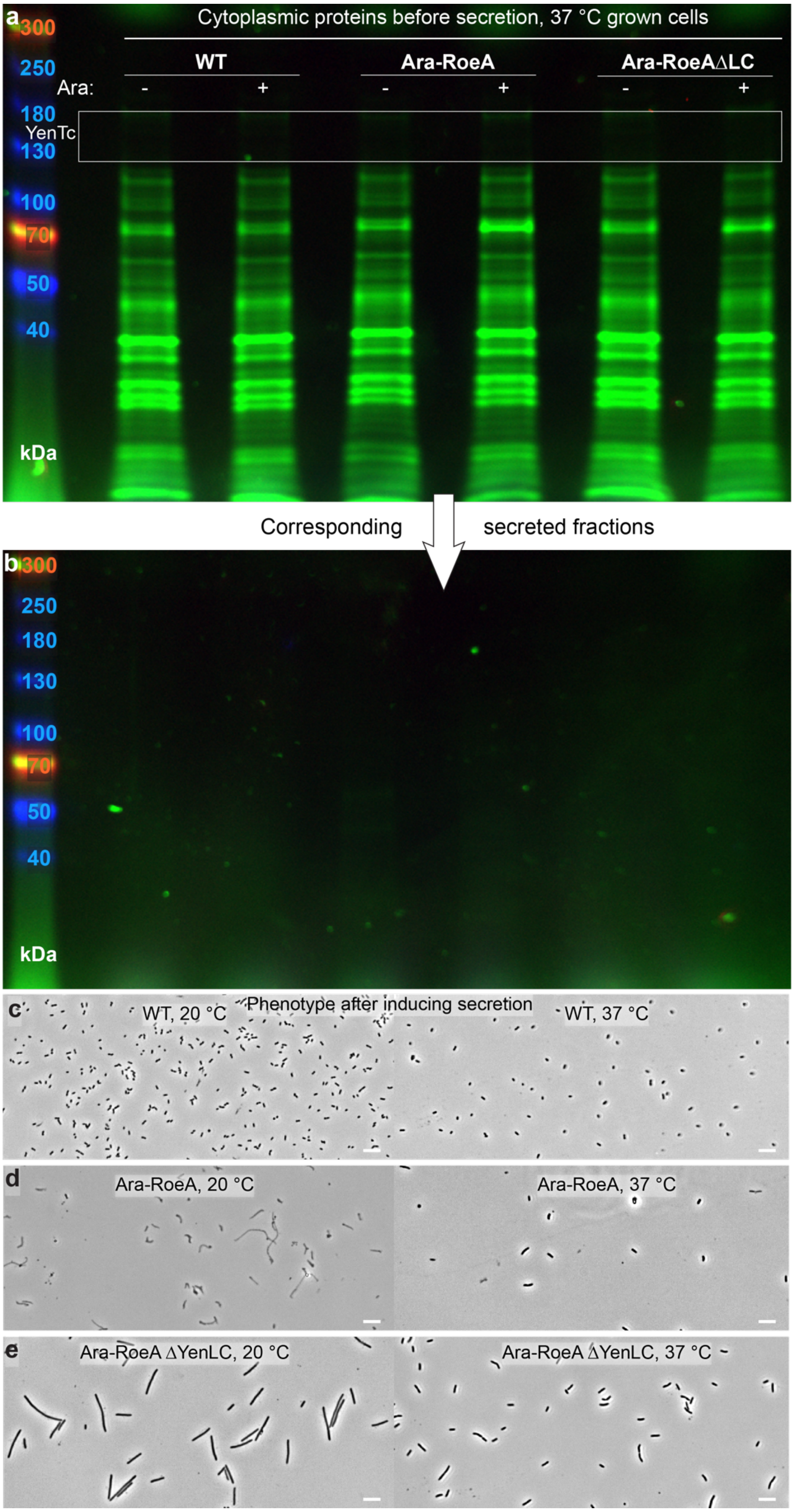

Supplementary Figure 3 | Soldier cell secretion and morphology is abolished at elevated temperatures. a-b, *Y. entomophaga* does not produce or secrete toxins when grown at 37 °C as opposed to the usual 20 °C, even when RoeA is induced to produce soldier cells. n = 3 biological replicates. **c-e**, The phenotypes of WT (**c**) and Ara-RoeA strain (**d**) cells grown at 20 °C and 37 °C after pH-induced secretion, with the Ara-RoeA ΔYenLC strain (**e**) serving as a negative control. Scale bars: 10 µm. n = 3 biological replicates.

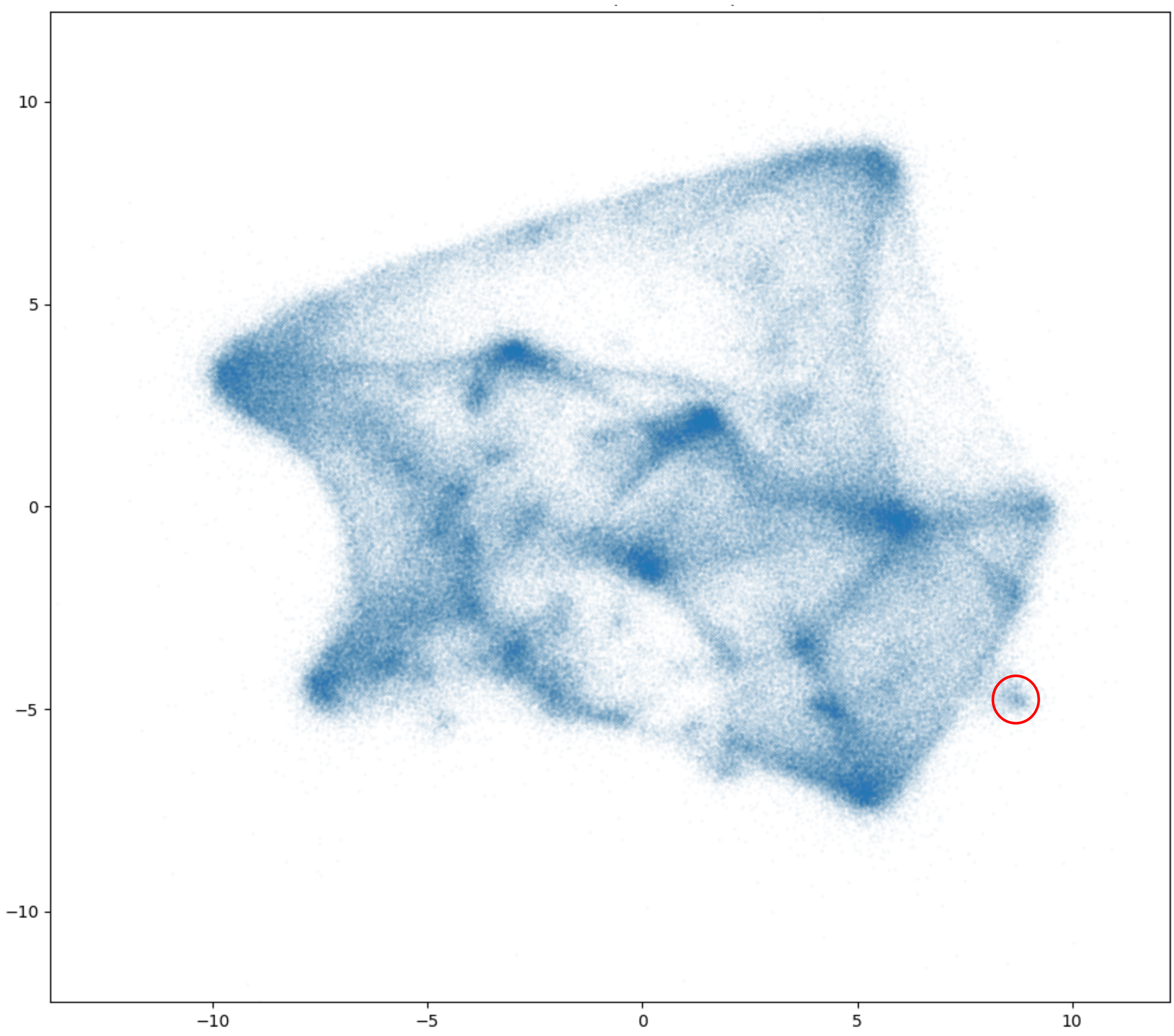

Supplementary Figure 4 | The YenTc cluster from a single embedded tomogram as seen in a TomoTwin UMAP. The UMAP was calculated on the median filtered embeddings, and average of all embedding vectors from this cluster was to create a reference embedding used for unsupervised picking of YenTc in the remaining tomograms.

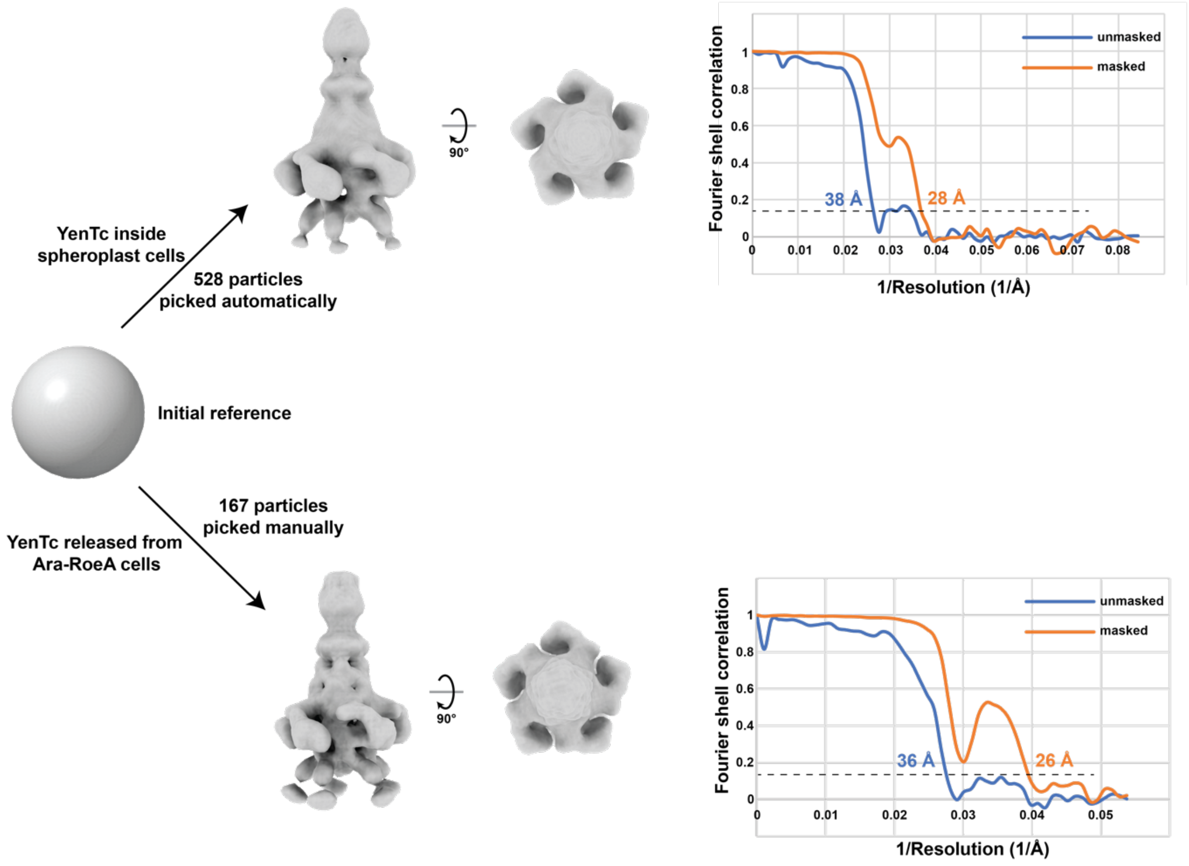

Supplementary Figure 5 | Overview of YenTc subtomogram averaging. Subtomogram averaging of YenTc localized intracellularly in Ara-RoeA ΔRz/Rz1 cells (top) and released from Ara-RoeA cells (bottom) with corresponding gold-standard FSC curves of the YenTc structures. The dip in the FSC curve at 33 Å corresponds to the first zero of the CTF curve at a defocus of -5.5 µm, which is the defoci of the majority of the particles.

Supplementary Table 1. *Y. entomophaga* strains and plasmids used in this study.

a. *Y. entomophaga* strains used in this study.

***Y. entomophaga* strain**	**Description**	**Source**
MH96	Wild type strain	German Collection of Microorganisms and Cell Cultures GmbH (DSMZ)
YenA1-sfGFP	sfGFP fusion after residue 37 of the YenA1 subunit of YenTc	This work
YenA2-sfGFP	sfGFP-His fusion to the C-terminus of the YenA2 subunit of YenTc + CmR	This work
YenA2-His	His-tag fusion to the C-terminus of the YenA2 subunit of YenTc	This work
RoeA-sfGFP	sfGFP fusion at the C-terminus of RoeA	This work
ΔHolA	Allelic gene replacement of HolA with CmR	This work
ΔPepB	Allelic gene replacement of PepB with CmR	This work
ΔRz/Rz1	Allelic gene replacement of Rz and Rz1 with CmR	This work
ΔYenLC	Allelic gene replacement of HolA, PepB, Rz and Rz1 with CmR	This work
Ara-RoeA	araC and AraBAD promoter inserted directly before RoeA	This work
Ara-RoeA ΔYenLC	Ara-RoeA and ΔYenLC combination strain	This work
Ara-RoeA ΔHolA	Ara-RoeA and ΔHolA combination strain	This work
Ara-RoeA ΔPepB	Ara-RoeA and ΔPepB combination strain	This work
Ara-RoeA ΔRz/Rz1	Ara-RoeA and ΔRz/Rz1 combination strain	This work
Ara-RoeA HolA-mCherry ΔPepB/Rz/Rz1	Ara-RoeA combination strain with HolA an mCherry fusion after a 30 aa linker, plus ΔPepB/Rz/Rz1	This work
Ara-RoeA YenA1-sfGFP	Ara-RoeA and YenA1-sfGFP combination strain	This work
ΔTat	Allelic gene replacement of TatA-TatD with CmR	This work
ΔT1SS	Allelic gene replacement of TolC with CmR	This work
ΔT2SS	Allelic gene replacement of PL78_RS08960-PL78_RS08990 with CmR	This work

ΔT3SS #1	Allelic gene replacement of PL78_RS19995-PL78_RS1969 with CmR	This work
ΔT3SS #2	Allelic gene replacement of PL78_RS18105-PL78_RS18250 with CmR	This work
ΔT6SS	Allelic gene replacement of PL78_RS00905-PL78_RS19390 with CmR	This work
ΔGshB	Allelic gene replacement of GshB with CmR	This work
ΔOxyR	Allelic gene replacement of OxyR with CmR	This work
ΔLipase #1	Allelic gene replacement of PL78_RS09630 with CmR	This work
ΔLipase #2	Allelic gene replacement of PL78_RS18445 with CmR	This work
ΔLipase #3	Allelic gene replacement of PL78_RS18020 with CmR	This work
ΔLipase #4	Allelic gene replacement of PL78_RS00835 with CmR	This work
ΔYenTc	Allelic gene replacement of Chi1-YenC2 with CmR	This work
YenA1-sfGFP ΔAI1	YenA1-sfGFP and acyl-homoserine-lactone synthase allelic gene replacement with GmR combination strain	This work
YenA1-sfGFP ΔAI2	YenA1-sfGFP and S-ribosylhomocysteine lyase allelic gene replacement with AmpR combination strain	This work
YenA1-sfGFP ΔAI3	YenA1-sfGFP and L-threonine 3-dehydrogenase allelic gene replacement with TcR combination strain	This work

S. marcescens strains used in this study.

***S. marcescens* strain**	**Description**	**Source**
BS 303 (aka ATCC 13880)	Wild type strain	German Collection of Microorganisms and Cell Cultures GmbH (DSMZ)
Ara-ChiR	araC and AraBAD promoter inserted directly before ChiR	This work
ΔSmaLC	Allelic gene replacement of ChiW, ChiX, ChiY and ChiZ with CmR	This work

b. Plasmids used in this study.

**Plasmid**	**Description**	**Source**
Ara-PepB	Arabinose-inducible PepB	This work
Ara-ChiR	Arabinose-inducible ChiR	This work
pMultiEdit-v4	Helper plasmid encoding arabinose-inducible λ-RED proteins, IPTG-inducible I-SceI restrictase, rhamnose-inducible Cas9 and constitutively expressed anti-N20 gRNA. The N20 is a sequence that has minimal off-target specificity in *Y. entomophaga*.	This work
pDonor	SacB-containing donor backbone plasmid for targeted genomic editing of regions of interest.	This work

Supplementary data references

1. Brurberg, M.B., Eijsink, V.G., Haandrikman, A.J., Venema, G. & Nes, I.F. Chitinase B from Serratia marcescens BJL200 is exported to the periplasm without processing. *Microbiology (Reading)* **141 (Pt 1)**, 123-31 (1995).
