# Supplementary material for: Yersinia entomophaga Tc toxin is released by T10SS-dependent lysis of specialized cell subpopulations
Source: Nat Microbiol. 2024 Jan 18;9(2):390–404. doi: 10.1038/s41564-023-01571-z (PMC10847048; doi:10.1038/s41564-023-01571-z)
Supplement: Supplementary file 2 — Reporting Summary [file 41564_2023_1571_MOESM2_ESM.pdf]

nature portfolio

Corresponding author(s): Stefan Raunser

Last updated by author(s): Nov 15, 2023

Reporting Summary

Nature Portfolio wishes to improve the reproducibility of the work that we publish. This form provides structure for consistency and transparency in reporting. For further information on Nature Portfolio policies, see our Editorial Policies and the Editorial Policy Checklist.

Statistics

For all statistical analyses, confirm that the following items are present in the figure legend, table legend, main text, or Methods section.

n/a	Confirmed	
☐	☒	The exact sample size (*n*) for each experimental group/condition, given as a discrete number and unit of measurement
☐	☒	A statement on whether measurements were taken from distinct samples or whether the same sample was measured repeatedly
☐	☒	The statistical test(s) used AND whether they are one- or two-sided<br>*Only common tests should be described solely by name; describe more complex techniques in the Methods section.*
☐	☐	A description of all covariates tested
☐	☐	A description of any assumptions or corrections, such as tests of normality and adjustment for multiple comparisons
☐	☒	A full description of the statistical parameters including central tendency (e.g. means) or other basic estimates (e.g. regression coefficient) AND variation (e.g. standard deviation) or associated estimates of uncertainty (e.g. confidence intervals)
☐	☒	For null hypothesis testing, the test statistic (e.g. *F*, *t*, *r*) with confidence intervals, effect sizes, degrees of freedom and *P* value noted<br>*Give P values as exact values whenever suitable.*
☐	☐	For Bayesian analysis, information on the choice of priors and Markov chain Monte Carlo settings
☐	☐	For hierarchical and complex designs, identification of the appropriate level for tests and full reporting of outcomes
☐	☐	Estimates of effect sizes (e.g. Cohen's *d*, Pearson's *r*), indicating how they were calculated

Our web collection on statistics for biologists contains articles on many of the points above.

Software and code

Policy information about availability of computer code

Data collection	Cryo-ET: SerialEM 3.8.5, Biochemistry: Bio-Rad ImageLab 5.2.1, MS: Xcalibur 4.0.27.10
Data analysis	Cryo-ET: Warp 1.0.9, IMOD 4.10.28, RELION 3.0, EMAN2 2.91, cryoCARE, Dragonfly 2022.1, ChimeraX 1.4, TomoTwin 0.3. Biochemistry: GraphPad Prism 9. MS: MaxQuant 2.0.3.2, Perseus 1.6.14.0, VolcaNoseR. Bioinformatics: SignalP 6.0, WebLogo 3.7.12, Clustal Omega, Ray Meta, CSAR-Web.

For manuscripts utilizing custom algorithms or software that are central to the research but not yet described in published literature, software must be made available to editors and reviewers. We strongly encourage code deposition in a community repository (e.g. GitHub). See the Nature Portfolio guidelines for submitting code & software for further information.

Data

Policy information about availability of data

All manuscripts must include a data availability statement. This statement should provide the following information, where applicable:

- Accession codes, unique identifiers, or web links for publicly available datasets
- A description of any restrictions on data availability
- For clinical datasets or third party data, please ensure that the statement adheres to our policy

Source data (includes unprocessed SDS-PAGE gels, mass spectrometry proteomics data, and raw data for graphs) are provided with this paper in the Source Data and Supplementary Source Data files. The raw mass spectrometry proteomics data have been deposited to the ProteomeXchange Consortium (https://

www.proteomexchange.org/) via the MassIVE partner repository with the dataset identifiers MSV00089961 / PXD035561 (secreted fraction vs. non-secreted fraction of Y. entomophaga cultures), MSV000089964 / PXD035573 (induced Ara-RoeA vs. WT or vs. non-induced Ara-RoeA Y. entomophaga), and MSV000091191 / PXD039813 (secreted fraction / pre-secretion fraction of induced S. marcescens Ara-ChiR vs. S. marcescens WT or vs. induced ΔSmaLC S. marcescens). YenTc cryo-ET structures from the post-endolysin and post-spanin states have been deposited in the Electron Microscopy Data Bank (https://www.ebi.ac.uk/emdb/) under accession numbers EMD-16618 and EMD-15403, respectively. Representative tomograms for Y. entomophaga are deposited under accession numbers EMD-15404 (pre-secretion state), EMD-15405 (post-holin state, FIB-milled), EMD-16619 (post-holin state, intact cells), EMD-15406 (post-endolysin state) and EMD-15407 (post-spanin state). Representative tomograms for S. marcescens in the post-spanin state are deposited under accession numbers EMD-16538 and EMD-16539. A conversion of our original strain nomenclature used during data deposition (July 2022) to the one adopted in this manuscript during review has been provided in the Supplementary Source Data file for ease of interpretation. No custom code was used in the analysis of the data. All biological materials are available from the authors upon request without restrictions. The UniProt database (https://www.uniprot.org/) was also used in this study.

Research involving human participants, their data, or biological material

Policy information about studies with human participants or human data. See also policy information about sex, gender (identity/presentation), and sexual orientation and race, ethnicity and racism.

Reporting on sex and gender	N/A
Reporting on race, ethnicity, or other socially relevant groupings	N/A
Population characteristics	N/A
Recruitment	N/A
Ethics oversight	N/A

Note that full information on the approval of the study protocol must also be provided in the manuscript.

Field-specific reporting

Please select the one below that is the best fit for your research. If you are not sure, read the appropriate sections before making your selection.

☒ Life sciences ☐ Behavioural & social sciences ☐ Ecological, evolutionary & environmental sciences

For a reference copy of the document with all sections, see nature.com/documents/nr-reporting-summary-flat.pdf

Life sciences study design

All studies must disclose on these points even when the disclosure is negative.

Sample size	No statistical methods were used to pre-determine sample sizes but our sample sizes are similar to those reported in previous publications.
Data exclusions	No data exclusions were made.
Replication	All experiments were carried out at least in biological triplicates.
Randomization	For the 3D refinement of cryo-EM/ET structures, particles were randomly split into two half sets. For all other experiments, randomization was not required because all data were used in the analysis.
Blinding	This study does not involve any experiments where blinding would be applicable.

Reporting for specific materials, systems and methods

We require information from authors about some types of materials, experimental systems and methods used in many studies. Here, indicate whether each material, system or method listed is relevant to your study. If you are not sure if a list item applies to your research, read the appropriate section before selecting a response.

Materials & experimental systems

n/a	Involved in the study
☒	☐ Antibodies
☒	☐ Eukaryotic cell lines
☒	☐ Palaeontology and archaeology
☒	☐ Animals and other organisms
☒	☐ Clinical data
☒	☐ Dual use research of concern
☒	☐ Plants

Methods

n/a	Involved in the study
☒	☐ ChIP-seq
☒	☐ Flow cytometry
☒	☐ MRI-based neuroimaging

Plants

Seed stocks

N/A

Novel plant genotypes

N/A

Authentication

N/A
